# Supplementary material for: The Tonoplast Topology Index—a new metric for describing vacuole organization
Source: Plant Methods. 2026 Jan 21;22:5. doi: 10.1186/s13007-025-01493-3 (PMC12821817; doi:10.1186/s13007-025-01493-3)

Additional File 1: A visual guide to interactive steps in TTI determination

This document contains additional examples illustrating user-modifiable steps in performing vacuole organization analysis using the TTI method, namely:

1. Algorithmic image processing
2. Selection of optical sections for VMI and TTI determination
3. Effect of image smoothing on detection of membrane crossings
4. Examples of effects of modifying peak detection parameters

1. Algorithmic image processing

This example shows a part of a single optical section from a confocal stack (benchmark image **top1a**) at successive stages of processing, documenting that topology of visible tonoplast membranes does not change during the processing steps. The first (raw) image is shown as it appears when opened with default settings of a fresh Fiji installation, which includes some automatic contrast adjustment for visualization (likely based on the first slice with little if any tonoplast signal), resulting in the "raw" image appearing overexposed.

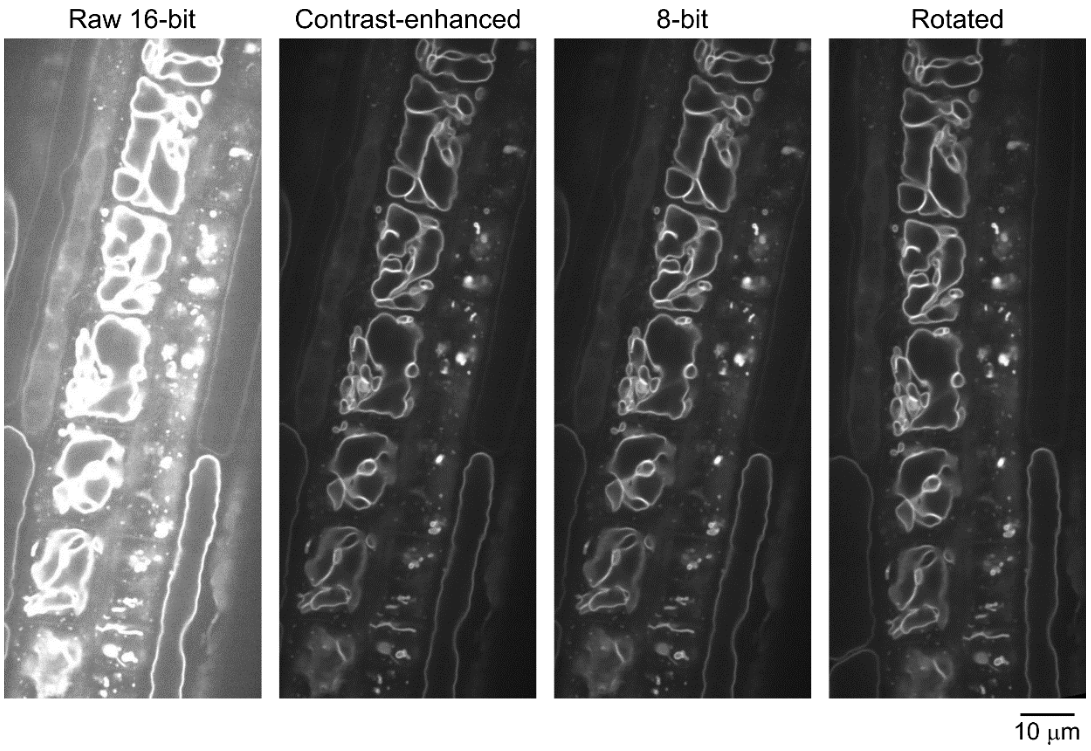

2. Selection of optical sections for VMI and TTI determination

Confocal optical section series of several representative cells from benchmark images **top4a** (top series) and **top5a** (bottom series) are shown. Sections (labelled in upper left corner of every image) were taken 1 µm apart, starting at the very cell cortex (section 1) and continuing through the tonoplast-free space occupied by the nucleus and perinuclear cytoplasm (marked by orange asterisks, located close to the cell wall adjacent to the cortex layer in elongation zone cells such as those shown). Cyan frames indicate all focal planes below the cell cortical zone and above the nucleus that can be used for VMI or TTI measurement, typically falling between 1/3 and 1/2 of the image stack´s Z-dimension (marked by section labels in magenta).

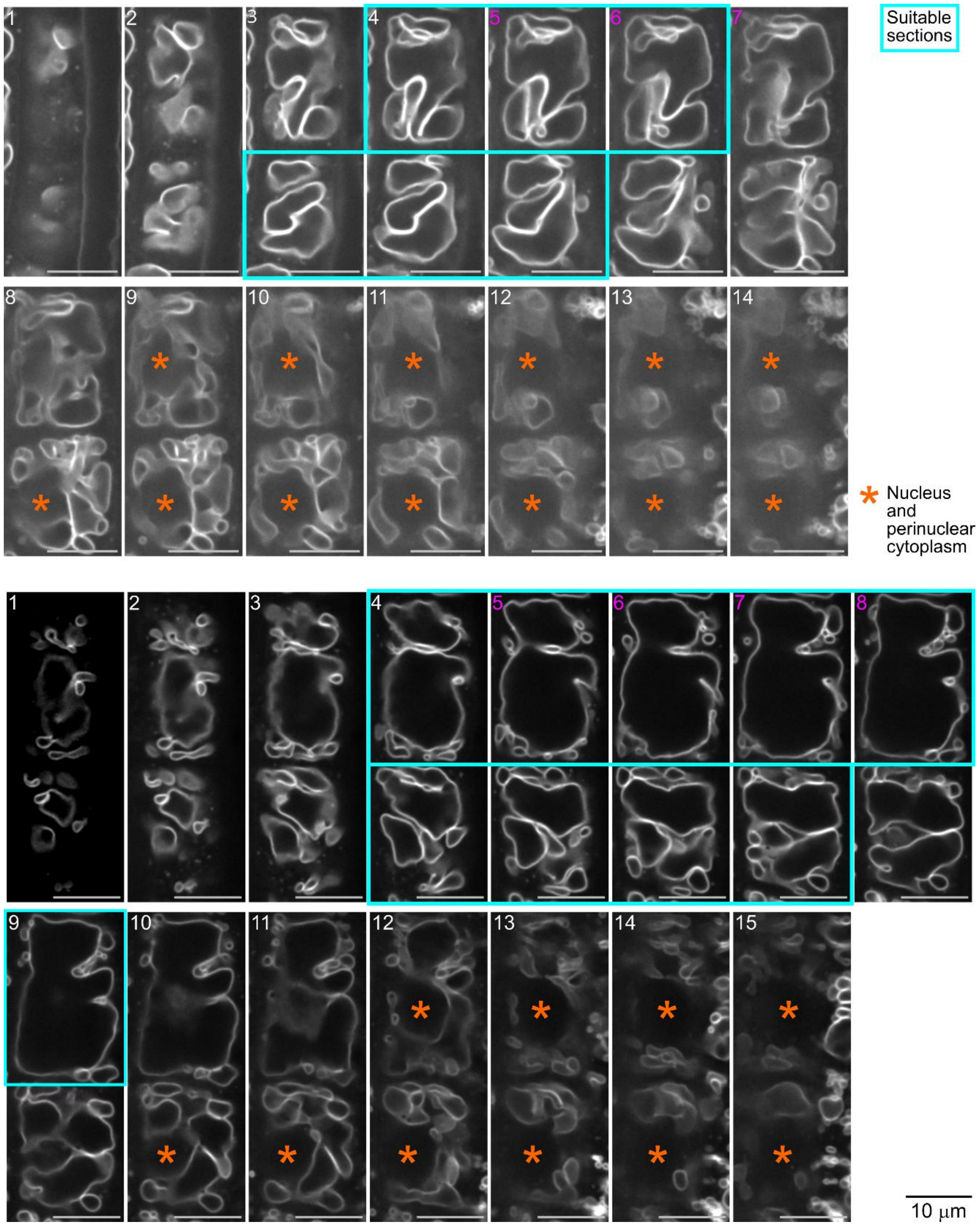

3. Effect of image smoothing on detection of membrane crossings

Examples of intensity profile graphs across the transect shown in cyan, obtained using the indicated number of smoothing cycles in the first (ImageJ) stage of the TTI determination procedure. Peaks corresponding to correctly identified membranes are denoted by arrowheads in cyan, probable artifacts (false peaks) in magenta, a missed peak in orange. The optical section originates from the benchmark image **top2a**.

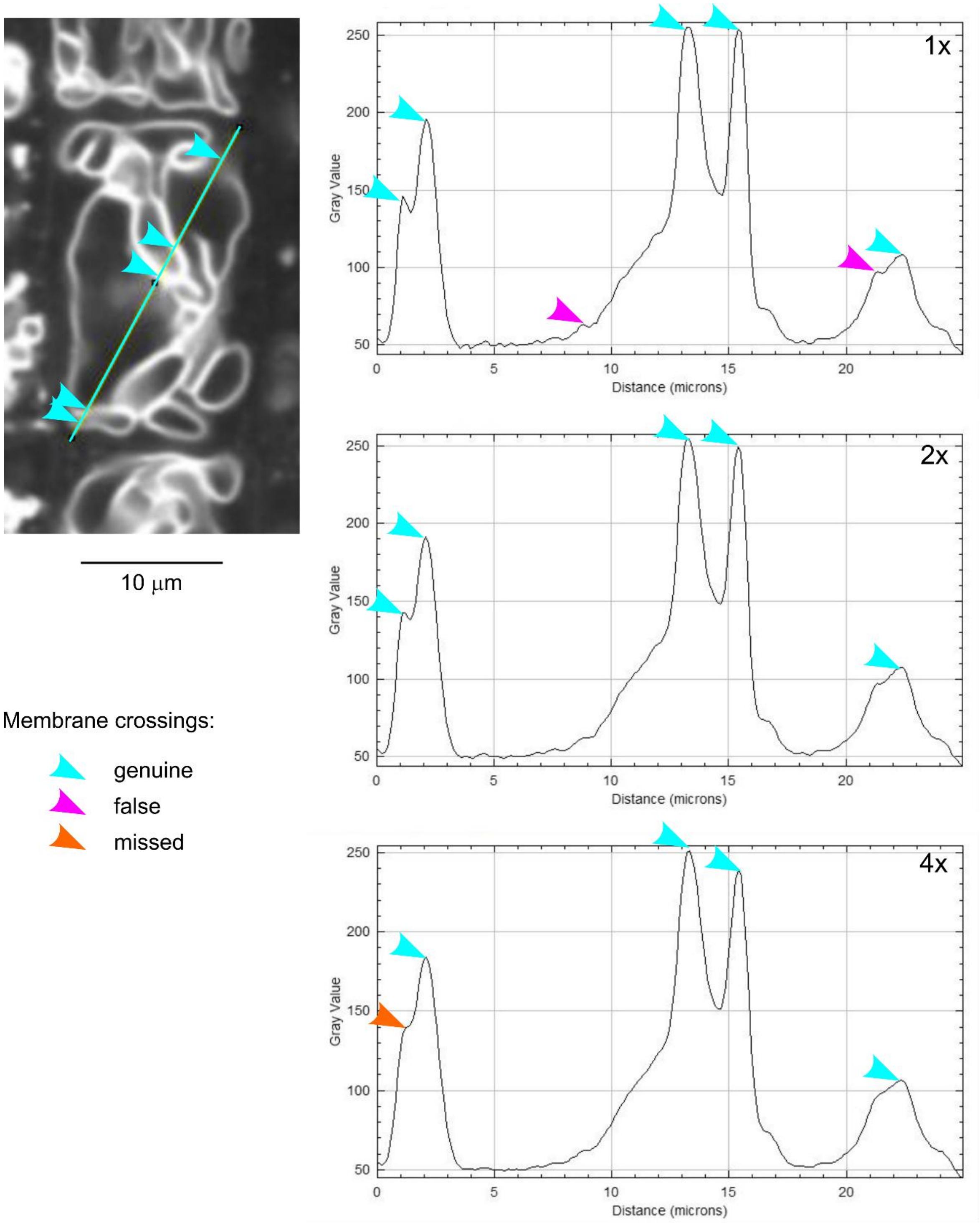

4. Examples of effects of modifying peak detection parameters

A sample optical section from image **top3a** with marked diagonal and longitudinal transects (in cyan for high TTI cells and in orange for a low TTI cell) is shown. Peak detection results using default Stage 2 parameters are provided for all cells, with additional examples of effects of modifying the adjustable parameters.

The default values are optimized for our typical microscopy images; thus, changes typically decrease detection efficiency (although raising the sigma value appears to be beneficial in this particular example). Minor parameter adjustments might not have noticeable effects (for example, raising background or prominence values up to 1 or the distance value up to 4 did not result in any changes for the sample data shown). However, different data (such as those from different hardware) may require parameter modification.

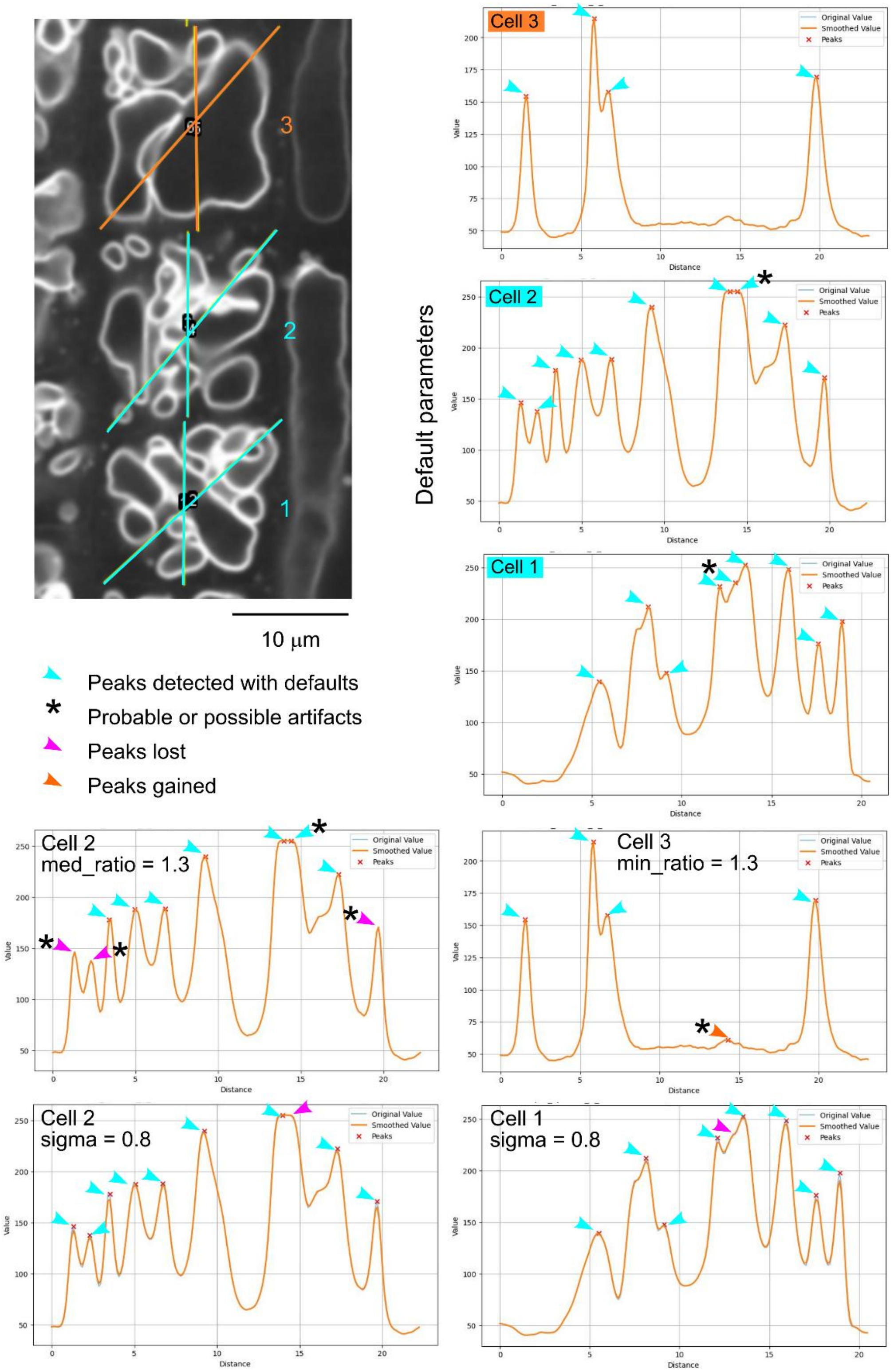

Supplement: Supplementary file 1 — Supplementary Material 1. A visual guide to interactive steps in TTI determination (*.pdf file). [file 13007_2025_1493_MOESM1_ESM.pdf]
